# Long-Term Oncological Outcomes for Young Men Undergoing Radical Prostatectomy for Localized Prostate Cancer

**DOI:** 10.1155/2017/9858923

**Published:** 2017-02-19

**Authors:** Daimantas Milonas, Zilvinas Venclovas, Inga Gudinaviciene, Kristina Zviniene, Aivaras Jonas Matjosaitis

**Affiliations:** ^1^Department of Urology, Lithuanian University of Health Sciences, Medical Academy, A. Mickeviciaus 9, LT-44307 Kaunas, Lithuania; ^2^Department of Pathology, Lithuanian University of Health Sciences, Medical Academy, A. Mickeviciaus 9, LT-44307 Kaunas, Lithuania; ^3^Department of Radiology, Lithuanian University of Health Sciences, Medical Academy, A. Mickeviciaus 9, LT-44307 Kaunas, Lithuania

## Abstract

*Aim*. The aim of this study was to describe PCa characteristics and long-term outcomes in young men aged ≤55 years after radical prostatectomy (RP) and to compare them with older men cohort.* Methods*. Among 2,200 patients who underwent RP for clinically localized PCa at our centre between 2001 and 2015, 277 (10.3%) men aged ≤55 years were identified. All preoperative and pathological parameters were compared between groups. Biochemical progression free survival (BPFS) and disease progression free survival (DPFS) were assessed at 5 and 10 years.* Results*. Men aged ≤55 years had similar pathological tumor characteristics and biochemical recurrence rate (BCR) compared to their older counterparts. Disease progression rate 2.5% versus 0.4% was higher in older patients (*p* = 0.026). BPFS rate was not different in both study groups. Estimated 10-year DPFS was 98.8% in younger men compared to 89.2% in their older counterparts (*p* = 0.031). Multivariate Cox regression showed that Gleason score lymph-nodes and surgical margins status were significant predictors for disease progression.* Conclusions*. In our cohort, men aged ≤55 years had similar pathological PCa characteristics and BCR rate in comparison with older men. RP can be performed with excellent long-term DPFS results in men with localized PCa at ≤55 years of age.

## 1. Introduction

Prostate cancer (PCa) is a disease of the elderly with 80% of men diagnosed at the age ≥65 [1]. However, PCa diagnosis is not uncommon in younger men, and the proportion of patients with PCa aged <50 years has increased from 1% in the 1970s to 5% in the PSA era [2]. Different autopsy studies show high rates of latent PCa in the fourth and fifth decades of age. The prevalence of latent PCa in younger men varies markedly among different autopsy series from 2.6% in Greek series [3] to a much higher 27% prevalence in Hungary [4] and up to 34% in USA [5]. Altogether, the study shows that about 20%–30% of 40–50-year-old men would harbor a PCa [6]. The increase of PCa diagnosis at young age raises a number of important questions about their biology and treatment modalities. A 60-year-old patient with a low risk and Gleason score 6 PCa is a suitable candidate for active surveillance. However, a similar scenario in men aged 45–55 might prompt immediate intervention in majority of cases, because of opinion that PCa detected in young age might behave more aggressively [7] and because young men are more likely to undergo RP [8]. Data about young age men with PCa treatment outcomes are controversial. According to the pre-PSA era studies, younger men are likely to have a more aggressive disease and carry a worse prognosis [9, 10]. However, more recent studies suggest high rates of indolent PCa with more favorable outcomes in young men after radical prostatectomy (RP) compared to their older counterparts [11–13]. A common limitation of these studies was prostate specific antigen (PSA) relapse used as endpoint of oncological outcomes, whereas disease progression or cancer related death should be the optimal endpoint in order to provide more generalizing conclusions.

The twofold aim of our study was to determine whether younger men had more favorable pathological findings and oncological outcomes (biochemical recurrence rate (BCR) and disease progression) in comparison with older counterparts and to evaluate prognostic risk factors for disease progression after RP.

## 2. Patients and Methods

Between 2001 and 2015, 2,200 men were treated with RP for clinically localized PCa at a single university hospital centre. We identified 277 men aged ≤55 years at time of RP. Clinical characteristics such as PSA level, clinical stage, and biopsy Gleason score were reported before RP. Pathological parameters (pathological stage, Gleason score, surgical margin status, and lymph nodes status) were collected after surgery. PSA testing after RP was performed every three months in the first year, biannually in the second and third year, and once a year thereafter. BCR was identified as a PSA value of >0.2 ng/ml in two consequent measurements. Disease progression was identified upon skeletal lesions confirmation by bone scan, CT, or MRI using RECIST criteria. Local recurrence was confirmed by histological investigation after surgery or biopsy. Pathological stage was assessed using 2002 TNM system, and tumor grading was classified using the Gleason grading system (2001–2005) and the revised 2005 Gleason grading system afterwards. Histopathological investigation in the majority of cases was performed by one uropathologist I.G. Adjuvant therapy (radiation therapy (RT) alone or RT + androgen deprivation therapy) was performed depending on the pathological characteristics of PCa within four months after RP, and salvage therapy (radiation therapy (RT) alone or RT + androgen deprivation therapy) was applied after detecting BCR. Prospective collection of data was approved by the university's ethical committee, and all patients signed a consent form provided before RP.

Clinical, pathological, and follow-up data (time to BCR, time to detected metastasis, or local recurrence) were compared between men aged ≤55 years at time of RP and older patients. The chi-square test for nominal variables and the *t*-test for continuous variables were used to compare baseline clinical and pathological characteristics. BPFS and DPFS rates for each study group were estimated using Kaplan-Meier analysis. The long-rank test was used to compare survival of younger versus older men. Men who underwent adjuvant therapy without BCR were excluded from BPFS analyses. The impact of baseline parameters on disease progression was assessed by bivariate and multivariate Cox regression analyses adjusted for clinical stage, preoperative PSA level, biopsy Gleason score, pathological stage, pathological Gleason score, lymph nodes, and surgical margins status. For this analysis PSA value was categorized to ≤4.0 versus 4.1–10 versus 10.1–20.0 versus >20.0 ng/ml.

## 3. Results

The number of men aged ≤55 years treated by RP in our centre increased significantly from 2.8% in 2003 to 15.5% in 2015. Within presented study cohort (Table 1), younger men aged ≤55 years were more likely to present with low PSA level (*p* = 0.038), clinically organ confined disease (*p* < 0.001), and less aggressive tumor according to biopsy Gleason score (*p* = 0.046). Pathological PCa stage (*p* = 0.1), grade (*p* = 0.37), and positive lymph nodes rate (*p* = 0.85) were not different comparing young men with older counterparts. BCR was similar between groups and reached 29% during median 50 months overall follow-up; metastases were detected at significantly higher rate in older men (*p* = 0.026). Skeletal disease progression lesions were detected in 40 of 50 (80%) cases, and in 10 (20%) cases local metastatic process was confirmed histologically after salvage surgery or biopsy. The median time to disease progression was 43 months in men aged >55 years versus 54 months in younger men.

**Figure 1:**
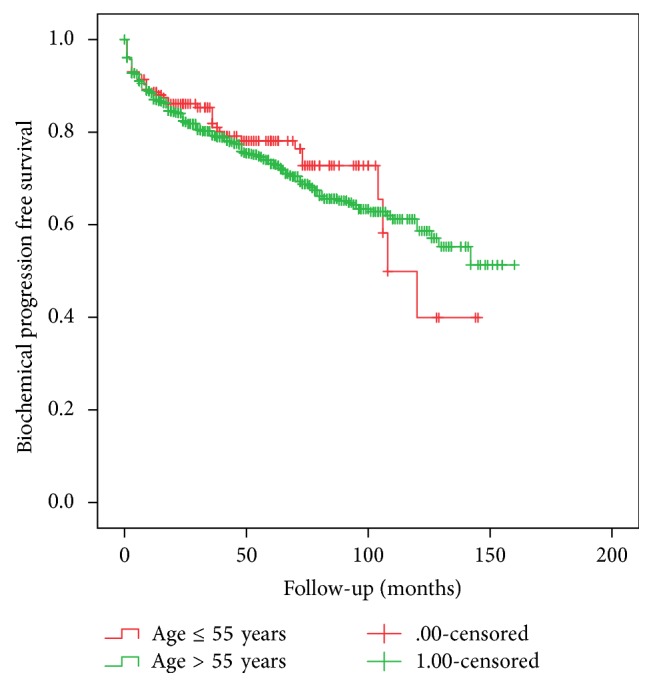
Comparison of Kaplan Meier biochemical progression free survival between study groups (log rank *p* = 0.57).

**Figure 2:**
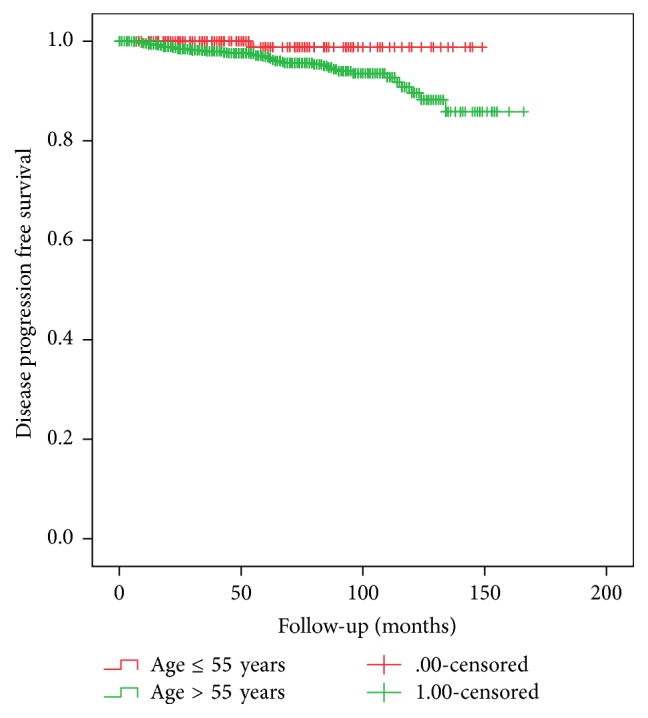
Comparison of Kaplan Meier disease progression free survival between study groups (log rank *p* = 0.031).

The Kaplan-Meier analysis showed similar BPFS rate (Figure 1) for men aged ≤55 years and older patients (2-, 5-, and 8-year BPFS was 85.9%, 77.9%, and 72.4% versus 82.8%, 73.7% and 63.7%, resp.; log rank *p* = 0.57); therefore different estimated DPFS rate (Figure 2) was detected between study groups (5- and 10-year DPFS was 98.8% and 98.8% versus 96.9% and 89.2% resp.; log rank *p* = 0.031).

Bivariate regression analysis revealed that almost all pre- and postoperative parameters, except patients' age and PSA below 20 ng/ml, are predictors for disease progression and Gleason score was most significant of them (Table 2). Multivariate Cox regression analysis shows that pathological Gleason score, positive surgical margins, and lymph nodes were mostly significant predictors of disease progression (Table 3). Patients' age (≤55 versus >55 years of age) failed to reach significant predictor status.

## 4. Discussion

Age at the detection of cancer is a well-recognized prognostic factor in a patient with majority localizations of malignancy. Although few studies demonstrated an association of a young age and high stage of PCa with worse prognosis [9, 10], data from recent studies have shown that earlier diagnosis of PCa in young men is associated with low grade and stage disease or even with superior outcome [11–13].

Becker et al. presented data of more than 13 thousand men who underwent RP at a single centre [13]. The authors compared men aged <50 and ≥50 years and detected a significant difference in pathological grade and stage between groups favorable to younger patients. Similar findings were presented by some other authors [11, 12]. Twiss et al. demonstrated opposite results in their analysis of 790 men after RP. These authors did not detect difference in preoperative and pathologic predictors of organ-confined disease and BCR between men aged <50 years and older [14]. Our data also showed that the proportion between organ-confined (70.4% versus 65.5%), locally advanced (29.6% versus 34.5%), low grade (Gleason score ≤ 3 + 4, 83.4% versus 80.6%), and high grade (Gleason score ≥ 4 + 3, 17.6% versus 19.4%) disease was similar when comparing young men and older counterparts, respectively. This suggests that data regarding pathological findings after RP in young men are still controversial. Therefore, data from recent studies confirm that pathological PCa characteristics in young men are not more aggressive than in older men.

Long-term BFSR for young men cohort presented in various studies is high. Becker et al. reported 80.7% and 63.0% estimated 5- and 10-year BFSR for men aged <50 years, and it was significantly higher (*p* = 0.006) compared to the older counterparts [13]. Freedland et al. presented 6-year BFSR data according to the decade of life in 1,753 men after RP and showed that men younger than 50 years of age had significantly higher BFSR compared to other groups [11]. Parker et al. also detected significantly lower BCR rate and highest BPFS among men aged <50 years versus all other age groups in their analysis of 5,195 men after RP [12]. In our study, 5- and 8-year BFSR was 77.9% and 72.4%, respectively, for young men, but the difference compared to men aged >55 years was not significant (*p* = 0.57). Looking at the data of all mentioned publications, we would like to emphasize that age at the time of surgery failed to achieve independent predictor status in multivariable analysis in most studies.

PSA relapse is not always associated with disease progression; therefore, behaviour of cancer could be estimated by disease progression or cancer related death. In present study, only one case (0.4%) of metastatic disease was detected in men aged ≤55 year compared to 49 cases (2.5%) in older men (*p* = 0.026). In presented cohort bivariate Cox proportional hazards model showed that clinical stage and biopsy Gleason score are significant predictors for disease progression, and the highest hazard ratio was detected for very poorly differentiated cancer (Gleason score 9-10). Preoperative PSA only at value >20 ng/ml could influence PCa progression. Younger patients age ≤55 years did not reach substantial level as independent parameter (*p* = 0.07). All pathological parameters in bivariate analysis were detected as highly significant predictors for disease progression (*p* = 0.001). Multivariate analysis shows that only some pathological parameters such as positive lymph nodes (HR 0.39, *p* = 0.002), positive surgical margins (HR 4.1, *p* = 0.001), and Gleason score (HR 2.4, *p* < 0.0001) are important for disease progression. These findings are in concordance with various other studies' data [15–20]. Interestingly enough, no difference was detected comparing these parameters between men aged ≤55 years versus >55 years in our cohort. Estimated 10-year DPFS was 98.8% versus 89.2% (*p* = 0.031) in favor of men aged ≤55 years. Presented data show that older men are more likely to progress to metastatic disease after a definitive treatment of BCR. More aggressive behavior of cancer in older men is unclear and needs more intensive multi-institutional research.

Two possible hypotheses arise trying to explain our study findings. The first one is that older men potentially have a more aggressive disease. Several studies comparing oncological outcomes after RP in young and older men supported such a conclusion [11–14]. In their review of young-age prostate cancer, Hussein et al. noticed that young-age PCa has several biological and genetic features that are distinct from elderly-onset cancer, but in the majority of cases young men tend to have low grade and stage disease. On the other hand, the authors pay attention that early-onset PCa could represent a subset of young-age and familial PCa with more aggressive disease and higher prostate-cancer-specific death rate [6]. Until now, only two factors (family history and race) are confirmed to have close relationship with the detection of PCa in young men [21, 22], but the data about its aggressiveness is controversial. We did not find more aggressive disease characteristics in younger men while comparing postoperative data, but higher disease progression rate in elderly patients directly supports the hypothesis that we have raised. The pathomechanism of such behavior is unclear and needs further research. Our second hypothesis is biologically age-related decreasing possibilities against disease progression and lower response of older patients to additional treatment after PSA relapse. No difference in grade, stage, lymph nodes involvement, and BCR rate logically suggests that additional conditions play an important role in disease progression. More long-term clinical data are needed to confirm our findings. In general, the results of present study regarding oncological outcomes indicate that young men with PCa could be suitable candidates for all treatment modalities.

The choice of treatment strategy depends not only on oncological outcomes, but also on the quality of life after definitive treatment. Continence and erectile function are most important parameters that concern men after the treatment they underwent. ProtectT trial regarding these two parameters shows significant inferiority of RP when compared to radiation therapy and active surveillance [23], and we should agree that RP harbors increasing risk of functional adverse events. Therefore, if we look at the data analyzing young men's population, continence end erectile function recovery rate is very high and reaches 95% and 80%, respectively, that is a significantly higher rate in comparison with older counterparts [13, 14]. The most important predictor for preservation of erectile function is nerve-sparing procedure [13, 14] that is strongly recommended in cases of young age and organ confided disease. So, we can conclude that men in young age with localized PCa are suitable candidates for surgical treatment with good DP control and low functional adverse events rate after RP.

The present study is not devoid of limitations. A relatively short follow-up is one of them. The absence of other treatment modality group and direct comparison of results is another limitation of our study. Although disease progression, not death from cancer, was chosen as the end point of this study, looking at our results we hope that the analysis of cancer specific mortality would show similar tendencies.

The strength of the present study is prospectively collected data, pathological investigation by one experienced pathologist in majority of cases, standard evaluation of disease progression, and treatment of BCR. To our knowledge, this study is the first one to describe disease progression free survival as end point in young patients cohort after RP.

## 5. Conclusions

The presented analysis of a large, single centre's cohort of men after RP indicates that young patients aged ≤55 years have similar histopathology and BCR rate after surgery for localized PCa compared to older counterparts. However, young patients have a significantly lower risk for disease progression in long-term follow-up and men aged ≤55 years with localized PCa should not be discouraged from radical treatment.

## Figures and Tables

**Table 1:** Clinical and pathologic features of men undergoing prostatectomy.

Parameter	Age ≤ 55 (*n* = 277)	Age > 55 (*n* = 1.923)	*p*
Follow-up (mo), median (quartiles)	48.5 (24.0–77.3)	50.0 (24.0–79.3)	0.8
PSA (ng/mL), median (quartiles)	5.8 (4.5–9.0)	6.36 (4.8–9.5)	0.038
Clinical stage, *n* (%)			
cT1	104 (37.5)	565 (29.4)	
cT2	154 (55.6)	1098 (57.1)	0.001
cT3	19 (6.9)	260 (13.5)	
Pathological stage, *n* (%)			
pT2	195 (70.4)	1260 (65.5)	
pT3a	70 (25.3)	519 (27.0)	0.1
pT3b	12 (4.3)	144 (7.5)	
Biopsy GS, *n* (%)			
3 + 3	183 (66.1)	1,125 (58.5)	
3 + 4	77 (27.8)	597 (31.0)	
4 + 3	10 (3.6)	75 (3.9)	0.046
8	5 (1.8)	87 (4.5)	
9-10	2 (0.7)	39 (2.0)	
Pathological GS, *n* (%)			
3 + 3	78 (28.2)	539 (28.0)	
3 + 4	153 (55.2)	1,011 (52.6)	
4 + 3	28 (10.1)	183 (9.5)	0.37
8	11 (4.0)	89 (4.6)	
9-10	7 (2.5)	101 (5.3)	
Pathological nodal status, *n* (%)			
N0	65 (23.5)	619 (32.2)	
N1	7 (2.5)	62 (3.2)	0.85
Surgical margins status, *n* (%)			
R0	214 (80.5)	1482 (80.1)	
R1	52 (19.5)	368 (19.9)	0.92
PSA relapse, *n* (%)	79 (28.5)	544 (28.3)	0.9
Disease progression, *n* (%)	1 (0.4)	49 (2.5)	0.026

PSA: prostate specific antigen, GS: Gleason score.

**Table 2:** Bivariate Cox proportional hazards analysis of factors predicting time to disease progression after radical prostatectomy.

Parameter	Hazard ratio	CI 95%	*p*
Clinical stage (cT):			
cT2 versus cT1	3.2	1.8–7.6	0.011
cT3 versus cT1	6.8	2.4–9.6	0.001
Pathological stage (pT):			
pT3a versus pT2	3.7	1.7–8.1	0.001
pT3b versus pT2	17.5	8.3–36.8	<0.001
PSA (ng/ml)			
4.1–10 versus ≤4	1.3	0.4–3.7	0.7
10.1–20 versus ≤4	2.1	0.7–6.4	0.2
>20 versus ≤4	5.3	1.6–16.9	0.005
Biopsy GS:			
3 + 4 versus 3 + 3	3.8	1.9–7.9	<0.001
4 + 3 versus 3 + 3	7.7	2.5–23.7	<0.001
4 + 4 versus 3 + 3	6.8	2.4–19.1	<0.001
≥4 + 5 versus 3 + 3	52.9	19.8–141.1	<0.001
Pathological GS:			
3 + 4 versus 3 + 3	N.D	N.D	N.D
4 + 3 versus 3 + 3	16.8	4.1–68.7	<0.001
4 + 4 versus 3 + 3	24.6	6.6–92.1	<0.001
≥4 + 5 versus 3 + 3	114.0	30.9–421.6	<0.001
Lymph nodes status (pN):			
N1 versus N0	4.5	2.0–9.9	0.001
Surgical margins:			
R1 versus R0	5.6	2.9–10.9	<0.001
Age, years			
≤55 versus >55	6.4	0.9–46.3	0.07

PSA: prostate specific antigen, GS: Gleason score.

**Table 3:** Multivariate Cox proportional hazards analysis of factors predicting time to disease progression after radical prostatectomy.

Parameter	Hazard ratio	CI 95%	*p*
Clinical stage	1.1	0.6–1.5	0.1
Pathological stage	1.2	0.7–2.0	0.6
PSA (ng/ml)	1.0	0.7–1.5	0.9
Biopsy GS	1.1	0.8–1.4	0.6
Pathological GS	2.4	1.7–3.1	<0.0001
Lymph nodes status			
N1 versus N0	0.39	0.15–0.65	0.002
Surgical margins			
R1 versus R0	4.1	1.8–9.4	0.001
Age, years			
≤55 versus >55	0.15	0.02–1.1	0.06

PSA: prostate specific antigen, GS: Gleason score.
